# Magnetic Resonance Imaging Correlates of White Matter Gliosis and Injury in Preterm Fetal Sheep Exposed to Progressive Systemic Inflammation

**DOI:** 10.3390/ijms21238891

**Published:** 2020-11-24

**Authors:** Robert Galinsky, Yohan van de Looij, Natasha Mitchell, Justin M. Dean, Simerdeep K. Dhillon, Kyohei Yamaguchi, Christopher A. Lear, Guido Wassink, Joanne O. Davidson, Fraser Nott, Valerie A. Zahra, Sharmony B. Kelly, Victoria J. King, Stéphane V. Sizonenko, Laura Bennet, Alistair J. Gunn

**Affiliations:** 1 Department of Physiology, University of Auckland, Auckland 1023, New Zealand; robert.galinsky@hudson.org.au (R.G.); nmit059@aucklanduni.ac.nz (N.M.); j.dean@auckland.ac.nz (J.M.D.); s.dhillon@auckland.ac.nz (S.K.D.); kyokyokyo330@gmail.com (K.Y.); christopher.lear@auckland.ac.nz (C.A.L.); g.wassink@auckland.ac.nz (G.W.); joanne.davidson@auckland.ac.nz (J.O.D.); v.king@auckland.ac.nz (V.J.K.); l.bennet@auckland.ac.nz (L.B.); 2 The Ritchie Centre, Hudson Institute of Medical Research, Melbourne, Victoria 3168, Australia; fraser.nott@hudson.org.au (F.N.); valerie.zahra@hudson.org.au (V.A.Z.); Sharmony.Kelly@monash.edu (S.B.K.); 3 Department of Obstetrics and Gynaecology, Monash University, Melbourne, Victoria 3800, Australia; 4 Division of Child Development & Growth, Department of Pediatrics, Gynaecology & Obstetrics, School of Medicine, University of Geneva, 1015 Geneva, Switzerland; yohan.vandelooij@epfl.ch (Y.v.d.L.); Stephane.Sizonenko@unige.ch (S.V.S.)

**Keywords:** preterm infant, inflammation, infection, brain, MRI

## Abstract

Progressive fetal infection/inflammation is strongly associated with neural injury after preterm birth. We aimed to test the hypotheses that progressively developing fetal inflammation leads to neuroinflammation and impaired white matter development and that the histopathological changes can be detected using high-field diffusion tensor magnetic resonance imaging (MRI). Chronically instrumented preterm fetal sheep at 0.7 of gestation were randomly assigned to receive intravenous saline (control; *n* = 6) or a progressive infusion of lipopolysaccharide (LPS, 200 ng intravenous over 24 h then doubled every 24 h for 5 days to induce fetal inflammation, *n* = 7). Sheep were killed 10 days after starting the infusions, for histology and high-field diffusion tensor MRI. Progressive LPS infusion was associated with increased circulating interleukin (IL)-6 concentrations and moderate increases in carotid artery perfusion and the frequency of electroencephalogram (EEG) activity (*p* < 0.05 vs. control). In the periventricular white matter, fractional anisotropy (FA) was increased, and orientation dispersion index (ODI) was reduced (*p* < 0.05 vs. control for both). Histologically, in the same brain region, LPS infusion increased microglial activation and astrocyte numbers and reduced the total number of oligodendrocytes with no change in myelination or numbers of immature/mature oligodendrocytes. Numbers of astrocytes in the periventricular white matter were correlated with increased FA and reduced ODI signal intensities. Astrocyte coherence was associated with increased FA. Moderate astrogliosis, but not loss of total oligodendrocytes, after progressive fetal inflammation can be detected with high-field diffusion tensor MRI.

## 1. Introduction

Preterm birth is associated with long-term neurodevelopmental impairments, such as cerebral palsy, for which the cumulative, lifetime economic cost in 2003 was estimated to be over USD11.5 billion [1]. More recent evidence suggests that the cost of disability associated with preterm birth continues to rise, and that disability prevention would significantly reduce economic burden on individuals, their families, and society [2,3]. The cause of neurodevelopmental impairment after preterm birth is multifactorial. However, there is compelling evidence that antenatal infection/inflammation is strongly associated with preterm birth and impaired neurodevelopment [4,5,6]. In recent cohort studies, long-term neurodevelopmental impairment after preterm birth was associated with diffuse injury in the white matter tracts, with evidence of chronic gliosis [7,8]. These pathological disturbances are considered to be key factors responsible for the combination of reduced white and grey matter volumes and long-term neurobehavioral and intellectual disabilities [9,10].

The etiology of neuroinflammation and related neuropathology is complex [6,11]. However, there is strong evidence that mild-to-moderate systemic inflammation is a major contributor to neural injury [6,12]. For example, in preterm infants, subclinical inflammation is associated with abnormal brain growth and neurological deficits without evidence of overt brain lesions [13,14,15,16]. Further, in preterm fetal sheep, low-dose intravenous lipopolysaccharide (LPS) infusion was associated with impaired maturation of electroencephalogram (EEG) activity and inflammation in the periventricular white matter and cortical grey matter [17]. In male newborn mice, twice-daily intraperitoneal injections of interleukin (IL)-1β for 5 days reduced myelination 30 days after the insult, as shown by regional reductions in fractional anisotropy and increased diffusivity using high-field diffusion tensor magnetic resonance imaging (MRI) [18].

While these data provide a compelling link between inflammation and impaired neurodevelopment, the most common clinical fetal and neonatal scenario involves a progressive inflammatory response that does not cause overt cardiovascular or placental compromise [19,20]. Furthermore, in fetal sheep, live bacterial inoculation at mid-gestation is associated with progressive systemic inflammation [21]. Similarly, recent evidence in preterm infants strongly suggests that fetal inflammation is a progressive process that persists into the neonatal period [22], supporting evidence that sustained inflammation after preterm birth is associated with long-term impaired brain development [23]. Thus, there is limited insight into the pathophysiology associated with neural injury during progressive fetal inflammation. Moreover, while MRI is widely used to define white matter injury after preterm birth, the relationship between MRI and histological changes after perinatal inflammation is poorly understood.

In the present study, we aimed to test the hypotheses that progressive systemic inflammation induced by the infusion of gram-negative LPS, without fetal hypotension, would lead to neuroinflammation and altered white matter development and that neuropathological changes associated with such progressive inflammation can be detected using diffusion-weighted MRI and neurite orientation dispersion and density imaging (NODDI). This study examined 0.7-gestation preterm fetal sheep, at an age when brain development is broadly equivalent to that of a preterm human infant at 28–30 weeks of gestation [24].

## 2. Results

### 2.1. Plasma Cytokines

**Figure 1:**
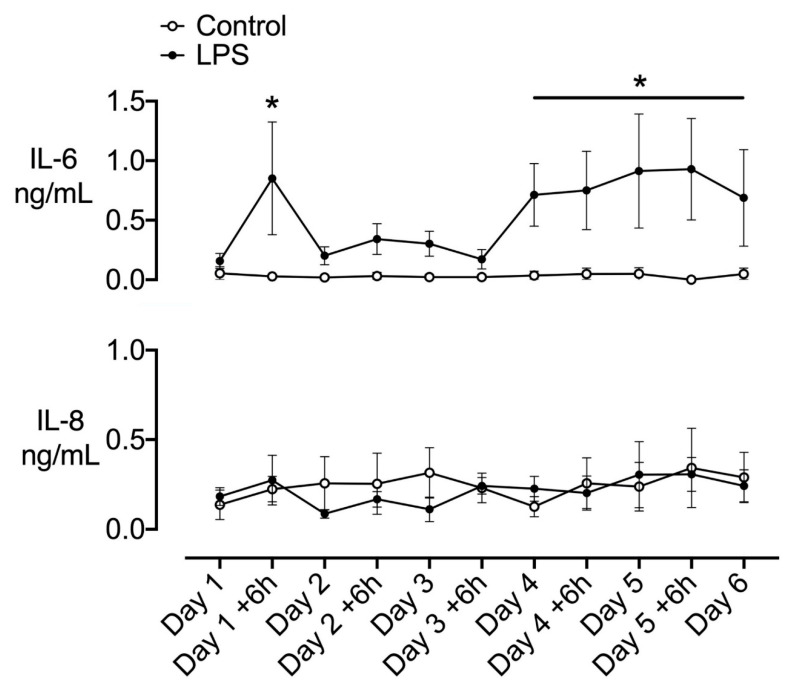
Time-course of changes in circulating interleukins. Time-course of changes in circulating interleukin (IL)-6 and IL-8 in the control (open circles, *n* = 6) and lipopolysaccharide (LPS) (closed circles, *n* = 7) groups. The dark shaded area represents the period of increasing LPS infusion. Data are hourly means ± SEM. * *p* < 0.05, LPS vs. control.

Circulating IL-6 levels were increased at +6 h (day 1) after starting LPS infusion (*p* < 0.05 vs. control, Figure 1). Levels transiently fell to near baseline levels on days 2 to 3 of LPS infusion but were elevated on days 4 and 5, and during the first day of recovery (day 6, *p* < 0.05 vs. control). Circulating IL-8 levels did not differ between groups (Figure 1).

### 2.2. Postmortem Findings

There were no significant differences in body weight, brain weight, and the numbers of males and females between the groups (Table 1).

### 2.3. Histopathology

#### 2.3.1. Gliosis

**Figure 2:**
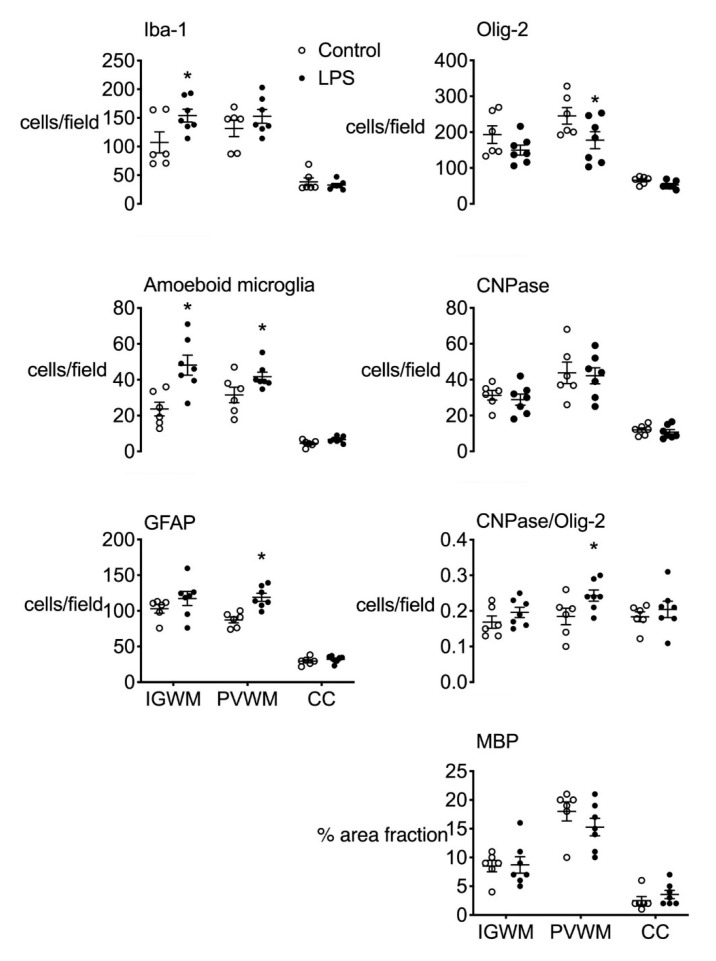
White matter immunohistochemistry. Ionized calcium-binding adaptor molecule 1 (Iba-1), oligodendrocyte transcriptase factor 2 (Olig-2), amoeboid (Iba-1 positive) microglia, and 2′,3′-cyclic-nucleotide 3′-phosphodiesterase (CNPase)-positive cell counts, the proportion of mature CNPase oligodendrocytes (CNPase/Olig-2 positive cells) and the percentage area of myelin basic protein (MBP)-positive staining in the intragyral and periventricular white matter tracts (IGWM and PVWM, respectively) and corpus callosum (CC) in control (open bars, *n* = 6) and LPS groups (closed bars, *n* = 7). Data are means ± SEM. LPS, lipopolysaccharide, * *p* < 0.05, LPS vs. control.

**Figure 3:**
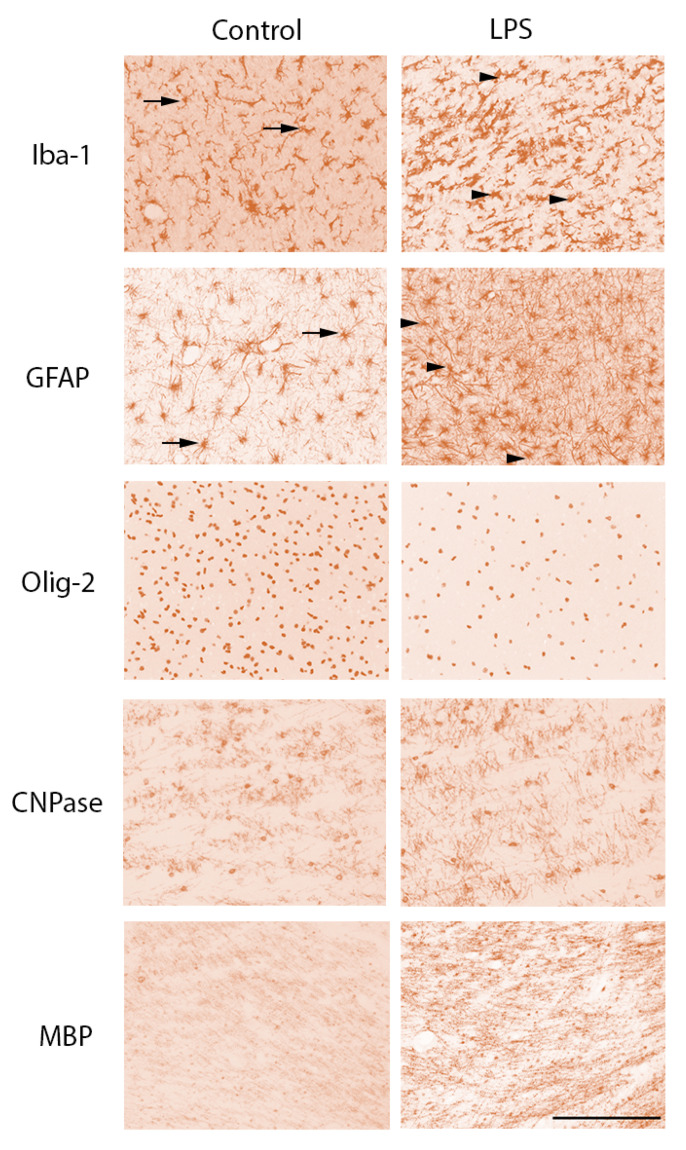
Photomicrographs of white matter tracts. Representative photomicrographs showing Iba-1-, glial fibrillary acidic protein (GFAP)-, Olig-2-, CNPase-, and MBP-positive staining in the periventricular white matter tracts. LPS, lipopolysaccharide. Scale bar is 200 µm. Arrows in the Iba-1 photomicrographs indicate microglia displaying a resting ramified phenotype, characterized by a small cell body with >1 branching process. Arrowheads in the Iba-1 photomicrographs from the LPS group indicate microglia displaying an amoeboid morphology, characterized by a large cell body with ≤1 branching process. Arrows in the GFAP photomicrographs indicate astrocytes with an isotropic appearance. Arrowheads in the GFAP photomicrographs from the LPS group indicate astrocytes displaying greater coherence and more anisotropy compared to controls.

The total number of ionized calcium-binding adaptor molecule 1 (Iba-1)-positive microglia and amoeboid microglia was increased in the intragyral white matter in the LPS group compared to control (*p* < 0.05, Figure 2). In the periventricular white matter, the number of amoeboid Iba-1+ microglia and glial fibrillary acidic protein (GFAP)-positive cells was increased in the LPS group compared to control (*p* < 0.05, Figure 2 and Figure 3). In the corpus callosum, there were no differences in the numbers of microglia or astrocytes between groups.

#### 2.3.2. White Matter Development

In the periventricular white matter, the number of oligodendrocyte transcriptase factor 2 (Olig-2)-positive cells (i.e., total oligodendrocytes) was reduced in LPS-exposed fetuses compared to control (*p* < 0.05, Figure 2 and Figure 3). The number of 2′,3′-cyclic-nucleotide 3′-phosphodiesterase (CNPase)-positive cells and area fraction of myelin basic protein (MBP) staining in the periventricular white matter did not differ between groups. The proportion of immature and mature (i.e., CNPase +/Olig-2+) oligodendrocytes was higher in LPS-exposed fetuses in the periventricular white matter compared control (*p* < 0.05, Figure 2). In the intragyral white matter and corpus callosum, the number of Olig-2- and CNPase-positive cells and area fraction of MBP staining did not differ between groups (Figure 2 and Figure 3).

### 2.4. Ex Vivo MRI

**Figure 4:**
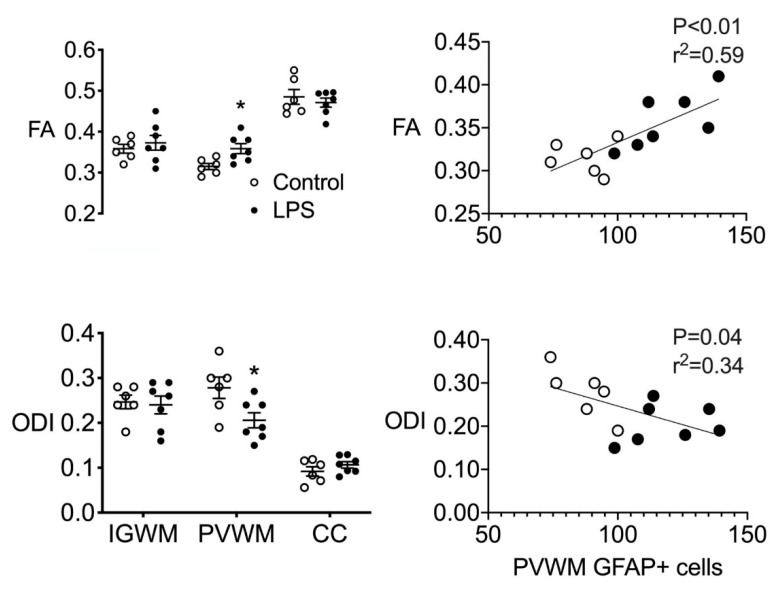
Diffusion tensor magnetic resonance imaging (MRI). (**Left**) Fractional anisotropy (FA) and orientation dispersion index (ODI) in the intragyral and periventricular white matter (IGWM and PVWM, respectively) and corpus callosum (CC) in control (open bars, *n* = 6) and LPS groups (closed bars, *n* = 7). (**Right**) Correlative analysis between FA, ODI, and periventricular GFAP-positive astrocytes in control (open circles, *n* = 6) and LPS groups (closed circles, *n* = 7). * *p* < 0.05, LPS vs. control.

Fractional anisotropy was increased, and orientation dispersion index was reduced in LPS-exposed fetuses in the periventricular white matter, compared to controls (*p* < 0.05, Figure 4). There were no differences in fractional anisotropy or orientation dispersion index in the intragyral white matter and corpus callosum between groups. There we no differences between groups for any of the other MRI parameters assessed.

### 2.5. Astrocyte Coherence Analysis

**Figure 5:**
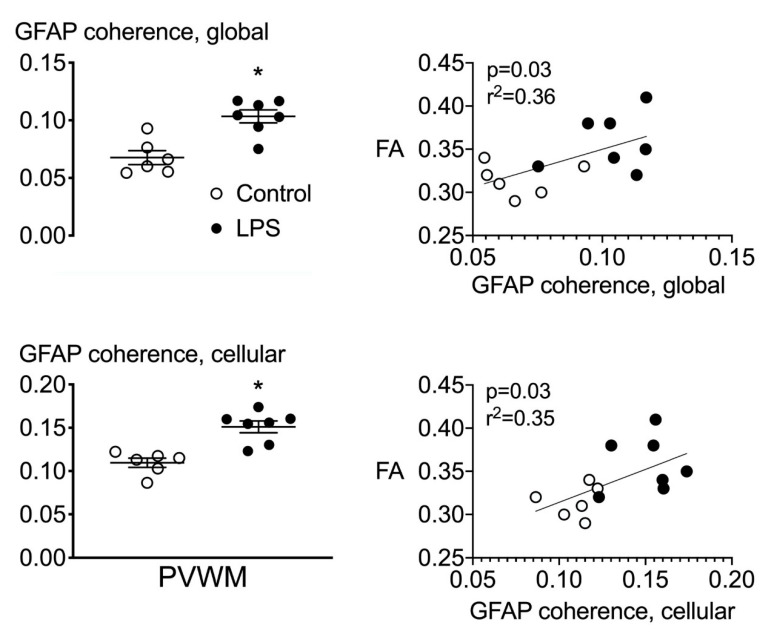
Astrocyte coherence. (**Left**) Astrocyte coherence in the periventricular white matter quantified from GFAP-stained histological sections. Global coherence was derived from whole periventricular white matter tissue sections. Cellular coherence represents coherence values derived from 15 randomly selected astrocytes per section. (**Right**) Correlative analyses between global and cellular coherence in the periventricular white matter from GFAP-stained tissue sections and fractional anisotropy. * *p* < 0.05, LPS vs. control.

The global coherence of GFAP-stained sections was increased in the periventricular white matter of the LPS-exposed group compared to control. Similarly, individual astrocyte coherence was increased in the periventricular white matter of the LPS group compared to control (*p* < 0.05, Figure 5).

### 2.6. Correlations

The intensities of fractional anisotropy and orientation dispersion index were associated with the total number of GFAP-positive cells in the periventricular white matter (linear regression: *p* < 0.01, r^2^ = 0.59 and *p* = 0.04, r^2^ = −0.34, respectively, Figure 4). There were no other significant correlations between measures of fractional anisotropy or orientation dispersion index and measures of microgliosis or myelination in the white matter regions that were examined. The intensity of fractional anisotropy was positively associated with the global coherence of GFAP-stained sections (*p* = 0.03, r^2^ = 0.36) and the coherence of individual astrocytes (cellular coherence, *p* = 0.03, r^2^ = 0.35, Figure 5). Physiologically, the spectral edge frequency of the EEG was positively associated with carotid artery blood flow (CaBF) (linear regression: *p* < 0.01, r^2^ = 0.53) and carotid artery vascular conductance (CaVC) (linear regression: *p* < 0.01, r^2^ = 0.48).

### 2.7. Fetal Biochemical and Physiological Outcomes

#### 2.7.1. Baseline Period

Before LPS/vehicle infusion, baseline mean arterial pressure (MAP), fetal heart rate (FHR), carotid and femoral blood flows, blood gases, and glucose and lactate concentrations were within the normal range by our laboratory standards and did not differ between groups (Table 2 and Supplementary Tables S1 and S2).

#### 2.7.2. Fetal Biochemistry

There were no significant differences in fetal arterial biochemistry parameters between groups during the baseline and infusion periods for pH, PaCO_2_, PaO_2_, glucose, and lactate. There was a small but significant increase in HCO_3_^-^ concentrations in the LPS group on days 2–4 compared to control (*p* < 0.05, Supplementary Table S1).

#### 2.7.3. Carotid and Femoral Arterial Blood Flows and Vascular Conductance

During LPS infusion, CaBF was higher in LPS-exposed fetuses on day 4 (*p* < 0.05 vs. control, Table 2). Similarly, carotid arterial vascular conductance was higher in LPS-exposed fetuses between days 4 and 5 (*p* < 0.05 vs. control, Table 2). Thereafter there were no differences in carotid arterial blood flow or vascular conductance between groups. Femoral artery blood flow (FBF) and vascular conductance (FVC) did not differ between groups throughout the experimental period (Supplementary Table S2).

#### 2.7.4. EEG Activity and Fetal Movement

EEG frequency was increased in LPS-exposed fetuses compared to that in controls between days 4 and 7 (*p* < 0.05 vs. control, Table 1). There were no differences in EEG power or fetal movement (assessed using nuchal EMG) between groups (Table 1).

#### 2.7.5. Fetal Heart Rate and Mean Arterial Pressure

During infusions, fetal heart rate (FHR) did not differ between groups. During the recovery period after the end of infusions, FHR was transiently higher in LPS exposed fetuses on days 8 and 10 (*p* < 0.05 vs. control for both time points, Supplementary Table S2). During LPS infusion, mean arterial blood pressure (MAP) was lower on days 3 and 4 (*p* < 0.05 vs. control for both time points, Supplementary Table S2). During the recovery period, there were no differences in MAP between groups.

## 3. Discussion

In the present study, progressive LPS-induced systemic inflammation increased gliosis and reduced total numbers of oligodendrocytes in the large white matter tracts of preterm fetal sheep but did not affect numbers of immature and mature oligodendrocytes or myelin expression. High-field (9.4T) diffusion tensor MRI showed that LPS exposure increased fractional anisotropy (FA) and reduced orientation dispersion index (ODI) signal intensities in the periventricular white matter. These MRI changes were closely correlated with astrogliosis but importantly, not with oligodendrocyte or myelin loss. Physiologically, this protocol of progressively increasing LPS infusion increased systemic cytokine production and increased EEG spectral edge frequency and carotid artery perfusion.

It is well established that inflammation plays a significant role in the pathogenesis of fetal and neonatal brain injury. However, there is compelling evidence that cases of fetal infection/inflammation associated with preterm birth and adverse neurodevelopmental outcomes are often relatively subtle and involve a sustained fetal and neonatal inflammatory response [12,22,23,25,26]. Even in cases of acute postnatal infection, on average, infants are not clinically diagnosed until a mean of 48 h after plasma cytokine levels begin to rise [27]. By contrast, most of the preclinical studies to date have focused on overt fetal infection/inflammation, typically using bolus doses of LPS or other infectious/inflammatory stimuli [28], and few studies have focused on the pathophysiological effects of prolonged inflammation [18]. Thus, preclinical models that are aligned with clinical experience are vital to improve our understanding of the pathophysiology of infection/inflammation-induced preterm brain injury and to help develop imaging techniques to facilitate early identification and treatment of neural inflammation.

In this study, we show that mild fetal inflammation, induced by progressive, systemic LPS infusion, was associated with prolonged upregulation of circulating interleukin (IL)-6 but had no effect on circulating IL-8. This differential effect on systemic cytokine induction is consistent with previous evidence in human neonatal and pediatric populations that demonstrates plasma IL-6 is a sensitive maker of systemic infection/inflammation [27,29,30,31].

Consistent with previous studies, progressive LPS-induced inflammation was associated with increased numbers of activated microglia and astrocytes in the white matter tracts [32,33,34]. The preterm brain is particularly vulnerable to microglia and astrocytes, likely due to their capacity to release pro-inflammatory mediators, including tumor necrosis factor and interleukin-1β, reactive oxygen species, and regulate intracellular calcium levels and ATP release during evolving neural injury [6,35]. The increased number of periventricular astrocytes observed in LPS-exposed fetuses is consistent with contemporary cases of diffuse white matter injury in preterm humans, in whom periventricular astrogliosis was tightly linked with impaired oligodendrocyte development [8].

We observed a significant reduction in the total number of oligodendrocytes (Olig-2+ cells) in LPS-exposed fetuses compared to that in controls in the periventricular white matter. In the preterm brain, it is well established that the periventricular white matter is highly susceptible to injury, however the underlying mechanisms responsible for this regional susceptibility to injury remain speculative. For example, in the preterm periventricular white matter there are vascular watershed zones that may predispose this area to more severe restrictions in substrate supply [36,37,38]. Speculatively, an inflammation-induced increase in cerebral metabolism may have increased susceptibility to reduced oxygen and substrate supply to the periventricular white matter of LPS-exposed fetuses. We observed no effect of fetal LPS infusion on myelination, with no differences in total numbers of immature and mature myelinating oligodendrocytes or MBP immunoreactivity between groups. However, there was an increase in the proportion of immature and mature (CNPase positive) oligodendrocytes in LPS-exposed fetuses, suggesting that the reduction in total oligodendrocyte number (fewer Olig-2-positive cells) was mediated by loss of oligodendrocyte progenitors, which have increased susceptibility to injury [39].

By contrast, in the corpus callosum, there were no differences in microglial and astrocyte numbers or oligodendrocyte survival between groups. The mechanism of this regional difference in response to systemic inflammation is unknown but is consistent with previous studies in adult and neonatal rodents exposed to inflammation that show limited effects of systemic inflammation on the corpus callosum [40,41].

Quantification of FA and ODI, using diffusion tensor MRI, is a powerful tool for examining microstructural changes during evolving injury in the developing brain [42]. However, the lack of direct quantitative comparisons between FA and ODI, and histological analyses has limited the interpretation of MRI findings. Further, a growing number of studies have shown added diagnostic value from high-field diffusion tensor MRI over lower-field-strength (3T) imaging [43,44]. In this study, using a 9.4T magnet, progressive fetal inflammation was associated with increased FA and reduced ODI in the periventricular white matter 10 days after starting the infusions. There was a positive linear relationship between FA and numbers of astrocytes in the periventricular white matter. Furthermore, in LPS-exposed fetuses, we observed an increase in astrocyte coherence (anisotropy) compared to that in the vehicle controls, and a positive linear relationship was observed between astrocyte coherence and FA. While MRI and histological analyses were performed on matched sections from contralateral hemispheres, in previous studies from our laboratory, we have consistently found similar changes in both hemispheres after systemic LPS exposure [17,32]. This is further supported by our observation of reduced anisotropy on both histological and MRI assessment in the current study.

Similar to our findings, after traumatic brain injury in adult rats, increased astrocyte numbers and astrocyte coherence were the main histological features associated with increased FA [45]. Orientation dispersion index was negatively associated with numbers of astrocytes in the periventricular white matter. In human postmortem tissue, astrocyte numbers contributed to the ODI values on diffusion MRI [46]. Conversely, increased FA and reduced ODI have been attributed to increased axonal thickness and myelination in preterm infants, possibly as part of a compensatory/reparative response to neural injury [47,48]. However, this is not supported by the present study, as there was no change in either the density of myelin or the number of immature and mature (CNPase positive) myelinating oligodendrocytes. The observation of increased FA within the white matter, instead of a decreased FA as is commonly observed at term equivalent after preterm birth, most likely relates to the temporal evolution of injury (for in-depth review see [49]). Our data strongly suggest that acute gliosis, observed 5 days after ending the LPS infusion, is a key cellular process that underlies the increase in FA. By contrast, clinically, reduced FA is observed months to years after preterm birth, and therefore, most likely reflects compensatory processes and structural abnormalities, such as delayed myelination and axonal injury [18,48,50,51]. Collectively, these data suggest that inflammation-induced changes in astrocyte number and morphology play an important role in modulating both the restriction and direction of water diffusion during evolving fetal neuroinflammation.

During LPS exposure, we observed an increase in EEG frequency, but not in power, that was not associated with changes in fetal oxygenation, acidosis, or carbon dioxide concentration. Carotid artery perfusion, a marker of cerebral blood flow [52], was either maintained or transiently increased, and so oxygen delivery was maintained or increased throughout. Intriguingly, the correlation between increased carotid artery perfusion and EEG frequency (r^2^ = 0.53) suggests the possibility that increased cerebral perfusion may have helped to support an inflammation-induced increase in brain metabolism. This concept is supported by studies in preterm infants that showed increased cerebral oxygen extraction after intrauterine inflammation and a greater incidence of peri/intraventricular hemorrhage in the first 24 h of life [53]. Similarly, studies in fetal sheep and newborn lambs showed intra-amniotic and intravenous LPS exposure was associated with increased carotid artery perfusion and oxygen delivery [32,54,55,56].

Thus, the inflammation-induced increase in carotid perfusion and frequency of neural activity may reflect changes in neuronal excitability. For example, astrocytes were increased in the present study in the white matter tracts after LPS exposure. Astrocytes have been shown to contribute to high-frequency neuronal activity through regulation of synaptic activity and neuronal excitability via gap junctions [57]. There is also evidence for direct neuronal effects. For example, increased expression of the chloride transporter, NKCC1, and a lower action potential threshold was reported in hippocampal pyramidal neurons from mice exposed to LPS at P14 [58]. Moreover, an increase in the frequency of α-amino-3-hydroxy-5-methyl-4-isoxazolepropionic acid (AMPA) receptor-mediated neuronal activity was observed in hippocampal neurons cultured with the pro-inflammatory cytokine interferon gamma [59]. Further studies are required to determine whether the effects of systemic inflammation on EEG activity are dependent on the magnitude of systemic inflammation and whether changes in neural function reflect inflammation-induced reprogramming of neural activity and/or metabolism.

Physiologically, LPS infusion was associated with a modest reduction in arterial blood pressure and did not produce overt changes in fetal heart rate, fetal movement, or placental function. The modest reduction in arterial blood pressure observed during LPS exposure in this study likely reflects the moderate level of systemic inflammation. For example, circulating IL-6 levels were approximately 6-fold lower compared to other studies in which acute systemic vasoconstriction followed by prolonged vasodilation developed after LPS exposure [32]. However, given that circulating IL-6 *per se* has been shown to have limited direct effects on blood pressure (for in-depth review see [60]), this raises the possibility that other pro-inflammatory pathways may be responsible for the modest reduction in blood pressure observed in this study. Speculatively, one mechanism of LPS-induced vasodilation is upregulation of inducible nitric oxide synthase (iNOS), which is a potent vasodilator [61,62]. Upregulation of iNOS plays a key role in vasodilation during septicemia and has been demonstrated after LPS exposure in fetal sheep [63]. There was no change in femoral artery perfusion, but there may have been vasodilation in other vascular beds, as shown by the increase in carotid artery perfusion and vascular conductance in a similar time course to the fall in arterial blood pressure (days 4–5). Collectively, these data demonstrate that the progressive, systemic LPS infusion produced a relatively mild circulating inflammatory response without overt signs of fetal distress or placental dysfunction. 

In conclusion, progressive fetal inflammation led to diffuse astrogliosis and reduced oligodendrocyte survival in the periventricular white matter 5 days after LPS infusion. For the first time, we show that increased fractional anisotropy and reduced orientation dispersion index in the periventricular white matter were closely correlated with astrogliosis and astrocyte coherence but, importantly, not with myelination or oligodendrocytes loss. Histological changes in white matter inflammation and development were associated with increased levels of circulating IL-6 and moderate increases in carotid artery perfusion and EEG activity, suggesting an inflammation-induced increase in neural metabolism and/or excitability. Based on these data, further studies are needed to assess the long-term impact of progressive inflammation on myelination and evaluate whether diffusion tensor MRI is capable of detecting improved histological outcomes associated with the treatment of fetal inflammation.

## 4. Materials and Methods

All procedures were approved by the Animal Ethics Committee of the University of Auckland under the New Zealand Animal Welfare Act and the Code of Ethical Conduct for animals in research established by the Ministry of Primary Industries, Government of New Zealand. This manuscript complies with the ARRIVE guidelines for reporting animal research [64]. Thirteen Romney/Suffolk fetal sheep underwent aseptic surgery between 97 and 99 days gestation (term = 147 days). Food but not water was withdrawn 18 h before surgery. Ewes were given long-acting oxytetracycline (20 mg/kg, Phoenix Pharm, Auckland, New Zealand) intramuscularly (i.m.) 30 min before the start of surgery. Anesthesia was induced by intravenous (i.v.) injection of propofol (5 mg/kg; AstraZeneca Limited, Auckland, New Zealand) and maintained using 2–3% isoflurane in O_2_ (Bomac Animal Health, NSW, Australia). During surgery, ewes received an i.v. infusion of isotonic saline (250 mL/h) to maintain fluid balance. Depth of anesthesia, maternal heart rate, and respiration were continuously monitored by trained anesthetic staff.

### 4.1. Instrumentation

In brief, following a maternal midline abdominal incision, the fetus was exposed, and polyvinyl catheters were inserted in the left femoral and brachial arteries, brachial vein, and amniotic cavity. Vascular flow probes (Transonic systems, Ithaca, NY, USA) were placed around the right carotid artery (3 mm) and right femoral artery (2.5 mm) to monitor carotid and femoral blood flows (CaBF and FBF, respectively). A pair of electrodes was sewn over the fetal chest to measure the fetal electrocardiogram (ECG). Two pairs of electroencephalograph (EEG) electrodes (AS633-7SSF; Cooner Wire, Chatsworth, CA, USA) were placed through burr holes onto the dura over the parasagittal parietal cortex (5 and 10 mm anterior to the bregma and 5 mm lateral) and secured with cyanoacrylate glue. A pair of electrodes was sewn into the nuchal muscle to record electromyographic (EMG) activity to measure fetal movement, and a reference electrode was sewn over the occiput. All fetal leads were exteriorized through the maternal flank. Antibiotics (Gentamicin; 80 mg; Rousell Ltd., Auckland, New Zealand) were administered into the amniotic sac before closure of the uterus. A maternal long saphenous vein was catheterized to provide access for postoperative care.

Sheep were housed in separate metabolic cages with access to water and food ad libitum in a temperature-controlled room (16 ± 1 °C, humidity 50% ± 10%) with a 12:12 h light/dark cycle. Five days of postoperative recovery were allowed before experiments commenced. During this time, ewes received intravenous antibiotics daily for 4 days (benzylpenicillin sodium, 600 mg; Novartis, Auckland, New Zealand and Gentamycin, 80 mg). Fetal catheters were maintained patent by continuous infusion of heparinized saline (20 IU/mL) at a rate of 0.2 mL/h.

### 4.2. Experimental Recordings

Fetal mean arterial blood pressure (MAP), corrected for maternal movement by subtraction of amniotic pressure, FBF, CaBF, ECG, EEG, and nuchal EMG was recorded continuously for offline analysis using custom data acquisition software (LabView for Windows, National Instruments, Austin, TX, USA). The blood pressure signal was collected at 64 Hz and low-pass filtered at 30 Hz. The fetal ECG was analog filtered between 0.05 and 100 Hz, digitized at 512 Hz, and used to derive fetal heart rate (FHR). The analog EEG signal was low-pass filtered with the cutoff frequency at 500 Hz, digitized at a sampling frequency of 1024 Hz, then further low-pass filtered (in software) with the low-pass cut off at 128 Hz, and down sampled to 256 Hz. The intensity (power) was derived from the intensity spectrum signal between 0.5 and 20 Hz, while the spectral edge was calculated as the frequency below which 90% of the intensity was present [65]. Total EEG power was normalized by log transformation (dB, 20× log intensity). The nuchal EMG signal was band-pass filtered between 100 Hz and 1 kHz, the signal was then integrated using a time constant of 1 s and digitized at 512 Hz.

### 4.3. Experimental Protocol

**Figure 6:**
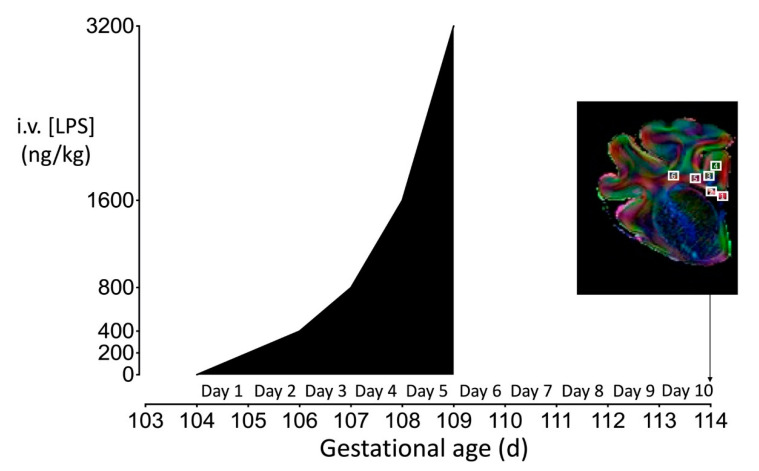
Schematic outlining the study design. The study consisted of two groups: control (*n* = 6), LPS (*n* = 7). The dark shaded area represents the period of progressive LPS infusion (200 ng LPS for the first 24 h, then doubled every 24 h for the next 96 h). Controls received an equivalent volume of vehicle (saline) during the infusion period. Continuous physiological recordings were performed throughout the experimental period. Fetal preductal arterial blood was collected every morning starting from 30 min before increasing the LPS or saline infusion and at 6 h after increasing the infusion for measurement of cytokine levels. Representative direction-encoded color map outlining the regions of interest sampled for MRI and histological analyses. Brain regions used for analysis were collected from the forebrain at the level of the mid-striatum from sections taken approximately 23 mm anterior to stereotaxic zero. White squares indicate regions of interest (ROIs) for assessment of the corpus callosum (1, 2), intragyral white matter within the first (3, 4) and the periventricular white matter (5, 6). The diffusion-weighted images and ROIs chosen for MRI analysis corresponded with the same regions used for histological assessment.

At 104 days, after 5–7 days postoperative recovery, fetuses were randomly allocated to receive an intravenous infusion of normal saline (*n* = 6) or lipopolysaccharide (LPS:*Escherichia coli,* 055:B5; *n* = 7) dissolved in saline. LPS was infused at 200 ng/kg (200 ng/mL at a rate of 41.7 µL/h) for the first 24 h, then doubled every 24 h for the next 96 h (Figure 6). The rationale for this LPS infusion protocol was to establish and sustain a fetal inflammatory response, as shown by the sustained increase in circulating fetal IL-6 concentration (Figure 1) without severe hypotension. Pilot studies carried out in our laboratory indicated this was the most effective and consistent protocol. Controls received the same volume of saline for infusion. Ten days after the start of infusions, sheep were killed by intravenous injection of an overdose (9 g) of pentobarbital sodium (Pentobarb 300, Chemstock international, Christchurch, New Zealand).

Fetal preductal arterial blood was collected every morning starting from 30 min before the experiment until the day of postmortem for pH, blood gases, (ABL 800, Radiometer, Copenhagen, Denmark), glucose, and lactate (model 2300, YSI, Yellow Springs, OH, USA).

### 4.4. Fetal Cytokine Measurements

Additional blood samples were collected immediately before the LPS or saline infusions and 2 and 6 h after starting infusion or changing the infusion rate, for measurement of cytokine levels using a commercially available enzyme-linked immunosorbent assay. The time points chosen for cytokine analysis were based on previous studies that used a similar experimental paradigm [32,66,67]. Plasma proteins (IL-6 and IL-8) were quantified using a sandwich enzyme-linked immunosorbent assay (ELISA). Flat-bottom 96-well plates (Nunc Maxisorp™; Thermo Fisher Scientific, Waltham, MA, USA) were coated with mouse anti-ovine IL-6 (1:200, cat# MCA1659 Bio-Rad Laboratories, Hercules, CA, USA) or mouse anti-ovine IL-8 (1:1000, cat# MCA1660, Bio-Rad) antibodies and incubated overnight at 4 °C. The next day, plasma samples were diluted with diluting buffer and incubated in duplicates in the 96-well plates for 1 h at room temperature. After washing, the plates were incubated with rabbit anti-ovine IL-6 (1:200, cat# AHP424, Bio-Rad) or rabbit anti-ovine IL-8 (1:4000, cat# AHP425, Bio-Rad) for 1 h at room temperature. Plates were then washed and incubated with horse radish peroxidase (HRP)-conjugated swine anti-rabbit l g (1:2000, DAKO, Santa Clara, CA, USA) for 1 h at room temperature. After further washing, plates were developed with 3.3′, 5.5′-tetramethylbenzidine (TMB chromogen solution; lnvitrogen, CA, USA) for 20 min in the dark at room temperature. Reactions were stopped with the addition of 0.5M H_2_SO_4_. The plates were read on a SpectraMax i3 microplate reader (Molecular Devices, San Jose, CA, USA) at 450 nm to determine optical density (OD). Standards (recombinant ovine IL-6 (cat# RP0367V-005) or IL-8 (cat# RP0488V-005); Kingfisher Biotech, St Paul, MN, USA) were included, and standard curves were generated for every ELISA plate used (R^2^ > 0.99 for all). Internal quality controls were included in each assay and cytokine concentrations were within the detection limit in all samples.

### 4.5. Histopathology

At postmortem (10 days after the start of infusions), fetal brains were perfusion fixed in situ with 0.9% saline solution followed by 10% phosphate-buffered formalin (1 L from 2 m height). Following removal from the skull, tissue was fixed for a further 5 days. The brain was sectioned through the sagittal midline, and the left hemisphere was prepared for ex vivo diffusion-weighted MRI, while the right hemisphere underwent processing and embedding using a standard paraffin tissue preparation. Brain slices were cut (10 μm thick) using a microtome (Leica Jung RM2035, Leica Microsystems, Albany, New Zealand). Brain regions of the forebrain used for analysis included the corpus callosum and periventricular and intragyral white matter from sections taken 23 mm anterior to stereotaxic zero. Slides were dewaxed in xylene, rehydrated in decreasing concentrations of ethanol, and then washed in 0.1 mol/L phosphate-buffered saline (PBS). Antigen retrieval was performed in citrate buffer (pH 6.0) using the pressure cooker technique in an antigen retrieval system (EMS Antigen 200 Retriever, Emgrid, Australia). Endogenous peroxidase quenching was performed by incubation in 0.1% H_2_O_2_ in methanol. Non-specific antigens were blocked using 3% normal goat serum. The sections were labeled with 1:200 rabbit anti-Olig-2 (Chemicon International; a marker of oligodendrocytes at all stages of the lineage) [68], 1:200 rabbit anti-Iba1 (Abcam, Hamilton, New Zealand), 1:200 mouse anti-GFAP (Abcam), 1:200 rabbit anti-CNPase (Abcam) and 1:200 rat anti-MBP (Merck, Dramstadt, Germany) overnight at 4 °C. Sections were incubated in biotin-conjugated IgG (1:200, goat anti rabbit, mouse or rat; Vector Laboratories, Burlingame, CA, USA) for 3 h at room temperature. Sections were incubated in ExtrAvidin^®^ (1:200, Sigma Aldrich, Auckland, New Zealand) for 2 h at room temperature and allowed to react with 3,3′-diaminobenzidine tetrahydrochloride (DAB; Sigma Aldrich). The reaction was stopped by washing in phosphate-buffered saline prior to being dehydrated and mounted.

**Figure 7:**
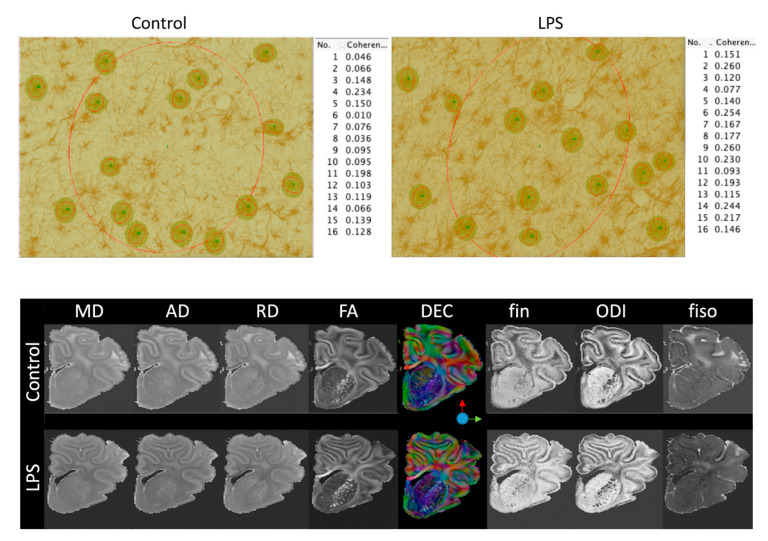
(**Top**) Representative examples of coherence analysis of GFAP-stained sections. Coherence analysis was performed using the OrientationJ plug-in for Fiji. Areas for coherence analysis were selected with the rectangular selection tool to measure coherence within the whole periventricular white matter section (global coherence, No. 1). The round selection tool was used to measure coherence for 15 individual astrocytes that were randomly selected from each periventricular white matter image (cellular coherence, no. 2–16). (**Bottom**) Mean diffusivity (MD), axial diffusivity (AD), radial diffusivity (RD), fractional anisotropy (FA); direction-encoded color (DEC) maps; intra-neurite volume fraction (fin); isotropic volume fraction (fiso); and orientation dispersion index (ODI). LPS, lipopolysaccharide.

Oligodendrocytes (Olig-2 and CNPase), myelination (MBP), microglia (Iba-1), and astrocytes (GFAP) were quantified at ×20 or ×40 magnification using a Nikon 80i light microscope and NIS elements BR 4.0 software (Nikon Instruments Inc., Melville, NY, USA). Total numbers of microglia (Iba-1-positive cells) and microglia displaying an amoeboid morphology were quantified, as previously described [69,70]. Astrocytes were quantified by counting the density of GFAP-positive cell bodies. Astrocytes that were deemed morphologically normal (GFAP-positive cells displaying a ramified morphology) and reactive (GFAP-positive cells with fewer and thicker retracted processes), as described by Liddelow et al. [71] were included. The area fraction of MBP immunoreactivity was determined with a standard intensity threshold using Fiji software (NIH, Bethesda, MD USA). Coherence analysis of GFAP-stained sections was performed using the OrientationJ plug-in for Fiji. OrientationJ characterizes the isotropic properties of a region of interest in an image based on the evaluation of the structure tensor. The OrientationJ measure tool was used to select the areas in Fiji to define the region through which OrientationJ creates the best fitting ellipse that represents the image gradient. Areas were selected with the rectangular selection tool to measure coherence within the whole periventricular white matter section (global coherence). The round selection tool was used to measure coherence for 15 individual astrocytes (cellular coherence) that were randomly selected from each periventricular white matter image (Figure 7). Average scores from the right hemisphere from four adjacent sections were calculated for each region and the region of interest (ROI, Figure 6) was randomized within each area. All imaging and cell counts were performed by an assessor who was blinded to the treatment.

### 4.6. Ex Vivo Magnetic Resonance Imaging

Fixed cerebral hemispheres (left hemisphere) were subjected to high-field diffusion tensor MRI using an actively shielded 9.4T/31 cm magnet (Agilent) equipped with 12 cm gradient coils (400 mT/m, 120 µs) and a 3.5 mm diameter birdcage coil. A multi-b-value shell protocol was used, based on a spin–echo sequence with the following parameters: field of view = 30 × 23 mm^2^, matrix size = 128 × 96, 20 slices of 1 mm thickness in the axial plane with a gap of 0.5 mm between each and 3 averages with echo time/repetition time = 45/3000 ms. A total of 96 diffusion-weighted images were acquired, 15 of them as b0 reference images. The remaining 81 were separated into 3 shells with the following distribution (number of directions/b-value in s/mm^2^): 21/1750, 30/3400, and 30/5100. All 81 directions were non-collinear and were uniformly distributed in each shell. The total acquisition time was 15 h/brain. Acquired data were fitted using a diffusion tensor imaging (DTI) toolkit (DTI-TK) to estimate DTI-derived parameters [72], and the neurite orientation dispersion and density imaging (NODDI) toolbox was used for NODDI estimates [73]. The ROIs were manually delineated on direction-encoded color maps to compute region of interest-averaged estimates of DTI and NODDI. Three specific white matter brain regions were identified: the periventricular white matter, intragyral white matter of the first parasagittal gyrus, and the corpus callosum (outlined in Figure 6). To ensure ROIs chosen for analysis were comparable between diffusion-weighted images and histological sections, both analyses were conducted on sections at the level of the forebrain at approximately 23 mm anterior to stereotaxic zero that included the corpus callosum and periventricular and intragyral white matter tracts. Prior to MRI analysis, brain regions were matched against histological sections by multiple assessors: R.G., J.M.D., and Y.v.d.L. Representative examples of the MRI parameters assessed are shown in Figure 7.

### 4.7. Data Analysis and Statistics

Offline physiological data analysis was performed using LabView-based customized programs (LabView for Windows). Carotid and femoral vascular conductance were calculated as mean blood flow/MAP. Due to a small but significant difference in baseline FHR (control: 192 ± 1 vs. LPS: 183 ± 1 bpm, *p* < 0.05), the relative change in FHR was calculated as the percentage change from the 24 h baseline period.

Statistical analysis was undertaken using SPSS (v22, Chicago, IL, USA) and Sigmaplot software (v12, Systat software, San Jose, CA, USA). Between- and within-group comparisons of fetal pH, blood gases, glucose, lactate, and physiological data were performed by two-way repeated-measures ANOVA. When statistical significance was found between groups or between group and time, post hoc comparisons were made using a Fisher’s least significant difference test. Between-groups comparisons of histological and MRI data were performed using a two-way ANOVA, followed by the Fisher’s least significant difference post hoc test when significance was found between groups or between region and group [74]. If there was an effect of region and group, the effect of group was assessed for each region separately. For coherence analysis, between-group comparisons were made using an unpaired t test. Mann–Whitney U-tests were used for testing non-parametric data. Linear and non-linear regressions were used as appropriate to compare the relationship between physiological variables, and between diffusion weighted MRI and markers of histological inflammation or injury. Post hoc power analysis for astrogliosis suggested 80% power to detect a minimum difference of 30 cells/field, with alpha of 0.05. Similarly, for oligodendrocyte survival, the study had 80% power to detect a minimum difference of 67 cells/field, with alpha of 0.05. Statistical significance was accepted when *p* < 0.05. Data are presented as mean ± SE.

## Figures and Tables

**Table 1:** Fetal body weight, brain weight, and sex.

	Body Weight (kg)	Brain Weight (g)	Sex (M, F)
Saline	2.01 ± 0.18	32.7 ± 1.4	2, 4
LPS	1.94 ± 0.13	31.1 ± 0.8	3, 4

Data are means ± SEM. LPS, lipopolysaccharide.

**Table 2:** Cerebrovascular and neurophysiological changes during fetal inflammation and recovery.

	CaBF (mL/min)	CaVC (mL/min/mmHg)	EEG (Hz)	EEG (dB)	Nuchal EMG (µV)
**Baseline**					
Control	28 ± 3	0.7 ± 0.1	10 ± 0	17 ± 1	2 ± 0
LPS	28 ± 4	0.8 ± 0.1	10 ± 1	16 ± 1	2 ± 0
**Day 1**					
Control	31 ± 2	0.8 ± 0.1	10 ± 0	17 ± 1	2 ± 0
LPS	31 ± 4	0.9 ± 0.1	10 ± 1	17 ± 1	2 ± 0
**Day 2**					
Control	28 ± 3	0.7 ± 0.1	10 ± 0	17 ± 1	2 ± 0
LPS	32 ± 5	0.9 ± 0.1	11 ± 0	17 ± 1	2 ± 0
**Day 3**					
Control	29 ± 3	0.7 ± 0.1	11 ± 0	18 ± 1	2 ± 0
LPS	36 ± 5	1.0 ± 0.1	11 ± 0	17 ± 1	2 ± 0
**Day 4**					
Control	29 ± 3	0.7 ± 0.1	10 ± 0	18 ± 1	2 ± 0
LPS	41 ± 5 *	1.2 ± 0.1 *	11 ± 0 *	17 ± 1	2 ± 0
**Day 5**					
Control	34 ± 2	0.8 ± 0.1	11 ± 0	18 ± 1	2 ± 0
LPS	44 ± 6	1.2 ± 0.2 *	12 ± 0 *	17 ± 1	2 ± 0
**Day 6**					
Control	37 ± 3	0.9 ± 0.1	11 ± 0	19 ± 1	2 ± 0
LPS	44 ± 6	1.2 ± 0.2	12 ± 0 *	17 ± 1	2 ± 0
**Day 7**					
Control	38 ± 3	0.9 ± 0.1	11 ± 1	19 ± 1	2 ± 0
LPS	43 ± 7	1.1 ± 0.2	12 ± 0 *	17 ± 1	2 ± 0
**Day 8**					
Control	38 ± 3	1.0 ± 0.1	11 ± 1	19 ± 1	2 ± 0
LPS	45 ± 8	1.2 ± 0.2	12 ± 0	18 ± 1	2 ± 0
**Day 9**					
Control	39 ± 3	1.0 ± 0.1	11 ± 1	19 ± 1	2 ± 0
LPS	46 ± 7	1.2 ± 0.2	12 ± 0	18 ± 1	2 ± 0
**Day 10**					
Control	41 ± 3	1.0 ± 0.1	11 ± 1	19 ± 1	2 ± 0
LPS	48 ± 7	1.2 ± 0.2	12 ± 0	18 ± 1	2 ± 0

Data are means ± SEM. LPS, lipopolysaccharide. CaBF, carotid artery blood flow; CaVC, carotid artery vascular conductance; EEG, electroencephalography; EMG, electromyography. * *p* < 0.05 vs. control.

## Supplementary Materials

Supplementary materials can be found at https://www.mdpi.com/1422-0067/21/23/8891/s1. Table S1, Arterial pH, blood gases, glucose and lactate values. Table S2. Cardiovascular changes during fetal inflammation and recovery.

## Abbreviations

CaBF	carotid arterial blood flow
CaVC	carotid arterial vascular conductance
CNPase	2′,-3′-cyclic-nuceotide 3′-phosphodiesterase
EEG	electroencephalogram
FBF	femoral arterial blood flow
FHR	fetal heart rate
FVC	femoral arterial vascular conductance
GFAP	glial fibrillary acidic protein
Iba-1	ionized calcium-binding adapter molecule 1
IGWM	intragyral white matter
IL	interleukin
LPS	lipopolysaccharide
MAP	mean arterial blood pressure
MBP	myelin basic protein
Olig-2	oligodendrocyte transcription factor 2
PBS	phosphate-buffered saline
PVWM	periventricular white matter
