# Supplementary material for: Magnetic Resonance Imaging Correlates of White Matter Gliosis and Injury in Preterm Fetal Sheep Exposed to Progressive Systemic Inflammation
Source: Int J Mol Sci. 2020 Nov 24;21(23):8891. doi: 10.3390/ijms21238891 (PMC7727662; doi:10.3390/ijms21238891)
Supplement: Supplementary file 1 [file ijms-21-08891-s001.pdf]

Supplementary table 1. Arterial pH, blood gases, glucose and lactate values.

		Day 1 baseline	Day 2	Day 2 +6h	Day 3	Day 3 +6h	Day 4	Day 4 +6h	Day 5	Day 5 +6h	Day 6 (recovery)
pH	Saline	7.33±0.01	7.34±0.01	7.33±0.01	7.33±0.01	7.33±0.01	7.33±0.01	7.32±0.01	7.33±0.01	7.32±0.01	7.33±0.01
	LPS	7.34±0.01	7.35±0.01	7.35±0.01	7.34±0.01	7.34±0.01	7.33±0.01	7.34±0.01	7.33±0.01	7.33±0.01	7.33±0.01
HCO_3^- (mmol/L)	Saline	23.0±0.6	23.2±1.0	23.4±0.7	23.3±0.7	21.7±1.3	22.9±0.6	22.9±0.8	22.9±0.7	23.1±0.7	24.1±0.6
	LPS	24.8±0.7	23.9±0.7	25.3±0.7*	24.5±0.5	23.8±0.4*	24.5±0.4	25.4±0.6*	23.3±0.6	23.9±0.4	25.0±0.6
PCO_2 (mmHg)	Saline	47.1±1.0	48.4±1.8	48.9±1.4	48.8±1.6	50.1±1.3	48.0±1.2	48.1±1.2	48.1±1.5	48.9±1.7	50.6±1.9
	LPS	50.1±1.4	49.5±1.0	50.5±1.2	49.9±1.1	49.0±1.1	52.2±1.2	52.1±1.0	49.3±1.6	49.4±1.1	52.2±1.4
PO_2 (mmHg)	Saline	22.6±1.2	23.2±1.4	21.9±1.3	23.7±1.2	22.1±1.4	23.5±1.2	22.0±1.3	24.2±1.4	21.8±1.2	24.0±1.1
	LPS	25.3±1.3	25.4±1.2	25.0±1.4	25.6±1.0	24.7±1.3	25.4±1.1	24.1±1.4	25.5±1.2	24.9±1.4	24.8±0.9
Glucose (mmol/L)	Saline	0.7±0.0	0.8±0.1	0.8±0.1	0.7±0.1	0.8±0.1	0.7±0.0	0.7±0.1	0.7±0.0	0.8±0.1	0.7±0.0
	LPS	0.8±0.0	0.9±0.1	0.9±0.1	0.8±0.1	0.9±0.1	0.8±0.0	0.9±0.1	0.8±0.0	0.8±0.1	0.9±0.1
Lactate (mmol/L)	Saline	0.7±0.1	0.7±0.0	0.9±0.1	0.8±0.0	0.8±0.0	0.8±0.0	0.9±0.1	0.8±0.0	0.9±0.1	0.8±0.0
	LPS	0.7±0.0	0.7±0.1	0.8±0.1	0.7±0.1	0.8±0.1	0.8±0.1	0.9±0.1	0.8±0.1	0.9±0.1	0.8±0.0

Data are means ± SEM. LPS, lipopolysaccharide, PCO2 partial pressure of arterial carbon dioxide, PO2 partial pressure of arterial oxygen, HCO_3^- bicarbonate. *P<0.05 vs control.

Supplementary table 2. Cardiovascular changes during fetal inflammation and recovery.

	FHR (% baseline)	MAP (mmHg)	FBF (mL/min)	FVC (mL/min/mmHg)
**Baseline**				
Control	100±0	37±1	13±3	0.3±0.1
LPS	100±0	36±1	11±2	0.3±0.1
**Day 1**				
Control	100±1	37±1	13±3	0.4±0.1
LPS	103±1	36±1	13±2	0.4±0.1
**Day 2**				
Control	99±1	37±1	14±3	0.4±0.1
LPS	100±1	36±1	12±2	0.3±0.1
**Day 3**				
Control	99±1	38±1	15±3	0.4±0.1
LPS	101±1	35±1*	13±3	0.4±0.1
**Day 4**				
Control	98±1	38±1	16±3	0.4±0.1
LPS	100±2	35±1*	15±3	0.4±0.1
**Day 5**				
Control	96±1	38±1	17±4	0.4±0.1
LPS	99±2	36±1	17±3	0.5±0.1
**Day 6**				
Control	95±2	38±1	19±4	0.5±0.1
LPS	98±1	37±1	17±3	0.5±0.1
**Day 7**				
Control	94±2	38±1	18±3	0.5±0.1

LPS	98±1	37±1	18±4	0.5±0.1
**Day 8**				
Control	93±2	38±1	20±3	0.5±0.1
LPS	98±2*	38±1	20±4	0.5±0.1
**Day 9**				
Control	93±2	39±1	22±4	0.6±0.1
LPS	96±1	38±1	21±4	0.5±0.1
**Day 10**				
Control	92±1	39±1	23±4	0.6±0.1
LPS	97±1*	39±1	23±4	0.6±0.1

Data are means ± SEM. LPS, lipopolysaccharide. FHR, fetal heart rate; MAP, mean arterial pressure; FBF, femoral blood flow; FVC, femoral vascular conductance.
*P<0.05 vs control.
